# Serological patterns of *Actinobacillus pleuropneumoniae*, *Mycoplasma hyopneumoniae*, *Pasteurella multocida* and *Streptococcus suis* in pig herds affected by pleuritis

**DOI:** 10.1186/s13028-016-0252-1

**Published:** 2016-10-04

**Authors:** Per Wallgren, Erik Nörregård, Benedicta Molander, Maria Persson, Carl-Johan Ehlorsson

**Affiliations:** 1 National Veterinary Institute, SVA, 751 89 Uppsala, Sweden; 2 Department of Clinical Sciences, Swedish University of Agricultural Sciences (SLU), Box 7054, 750 07 Uppsala, Sweden; 3 Farm & Animal Health, Kungsängens Gård, 753 23 Uppsala, Sweden

**Keywords:** Pig, Respiratory disease, Pleuritis, Antibodies, Disease pattern

## Abstract

**Background:**

Respiratory illness is traditionally regarded as the disease of the growing pig, and has historically mainly been associated to bacterial infections with focus on *Mycoplasma hyopneumoniae* and *Actinobacillus pleuropneumoniae*. These bacteria still are of great importance, but continuously increasing herd sizes have complicated the scenario and the influence of secondary invaders may have been increased. The aim of this study was to evaluate the presence of *A.* *pleuropneumoniae* and *M. hyopneumoniae*, as well as that of the secondary invaders *Pasteurella multocida* and *Streptococcus suis* by serology in four pig herds (A–D) using age segregated rearing systems with high incidences of pleuritic lesions at slaughter.

**Results:**

Pleuritic lesions registered at slaughter ranged from 20.5 to 33.1 % in the four herds. In herd A, the levels of serum antibodies to *A.* *pleuropneumoniae* exceeded A_450_ > 1.5, but not to any other microbe searched for. The seroconversion took place early during the fattening period. Similar levels of serum antibodies to *A.* *pleuropneumoniae* were also recorded in herd B, with a subsequent increase in levels of antibodies to *P.* *multocida*. Pigs seroconverted to both agents during the early phase of the fattening period. In herd C, pigs seroconverted to *P.* *multocida* during the early phase of the fattening period and thereafter to *A.* *pleuropneumoniae*. In herd D, the levels of antibodies to *P. multocida* exceeded A_450_ > 1.0 in absence (A_450_ < 0.5) of antibodies to *A.* *pleuropneumoniae*. The levels of serum antibodies to *M.* *hyopneumoniae* and to *S.* *suis* remained below A_450_ < 1.0 in all four herds. Pigs seroconverted to *M.* *hyopneumoniae* late during the rearing period (herd B–D), or not at all (herd A).

**Conclusion:**

Different serological patterns were found in the four herds with high levels of serum antibodies to *A.* *pleuropneumoniae* and *P.* *multocida*, either alone or in combination with each other. Seroconversion to *M.* *hyopneumoniae* late during the rearing period or not at all, confirmed the positive effect of age segregated rearing in preventing or delaying infections with *M.* *hyopneumoniae.* The results obtained highlight the necessity of diagnostic investigations to define the true disease pattern in herds with a high incidence of pleuritic lesions.

## Background

Respiratory illness is traditionally regarded as the disease of the growing pig, and has historically been associated with bacterial infections such as *Mycoplasma* *hyopneumoniae* [1–3] and *Actinobacillus* *pleuropneumoniae* [4–6]. These bacteria still are of great importance, but the continuously increasing herd sizes have complicated the clinical picture. As the number of transmission events between pigs in a population is equal to the number of pigs multiplied with the number of pigs minus one [x = n * (n − 1)], they will escalate as the herd size increase [7]. Thus, the number of transmission events between pigs will increase with a factor of around four if a population is doubled and with a factor of around 100 if a population is enlarged ten times.

The increased number of transmissions between pigs may increase the influence of other microbes. *M.* *hyopneumoniae* and *A.* *pleuropneumoniae* are important pathogenic microbes, but co-infections may intensify or prolong clinical signs of respiratory disease [8–11]. It has also been observed that the incidence of respiratory illness may vary with season [12]. Therefore, infections in the respiratory tract of grower pigs have become regarded as a syndrome rather than linked to single microorganisms [11, 13, 14]. This syndrome is referred to as the porcine respiratory disease complex (PRDC). As stated above PRDC is regarded to be dominated by bacterial species, and important primarily pathogenic bacterial species include *M.* *hyopneumoniae* [1–3] and *A.* *pleuropneumoniae* [4–6]. The frequent demonstration of interferon-α in serum in growers during the first week after arrival to fattening herds [15, 16] suggest that PRDC can be associated with viral infections, and that PRDC can also include the influence of secondary invaders such as *Pasteurella* spp [17, 18].

**Fig. 1:**
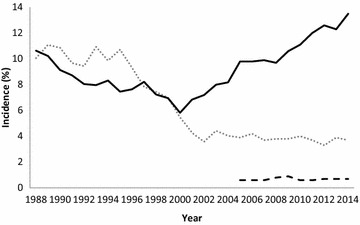
National incidence of pathological lesions in the respiratory tract of pigs in Sweden at slaughter during a period of 26 years [21]. The* figure* shows the annual incidence of respiratory lesions registered of the entire Swedish pig population. In 1980 around four million pigs were slaughtered. In 2014 around three million pigs were slaughtered. Pneumonia of mycoplasma type *dotted grey line*; Pneumonia of acute *A.* *pleuropneumoniae* type *dashed black line*; Pleuritis *black line*

When Sweden in 1986 as the first country in the world banned the use of low dose antibiotics in animal feed for growth promotion, some introductory health disturbances were recorded. As a consequence, a strict age segregated rearing from birth to slaughter was implemented in a large scale, which improved health as well as productivity [19, 20]. As seen in Fig. 1, the incidence of recorded pathogenic lesions in the respiratory tract at slaughter decreased during the last decade of the twentieth century [21]. The registrations of pneumonia at slaughter has remained stable at that level since then. In contrast, the incidence of recorded pleuritis at slaughter has continuously increased since the year 2000, as has the clinical evidence of actinobacillosis [22]. Discussions concerning the reason for this increase has included suggestions of introduction of new strains, or mutation of existing strains of *A.* *pleuropneumoniae*. However, acute actinobacillosis has in Sweden historically been dominated by serotype 2, and is still dominated by that serotype [22]. Further, Pulse Field Gel Electrophoreses has revealed that strains isolated in the twenty-first century were identical to strains isolated in the 1970s and 1980s [23]. As a consequence, the increase of actinobacillosis and pleuritic recordings at slaughter has merely been linked to the continuously increasing herd sizes with increasing number of transmissions of microbes between pigs, within and between units [22].

The aim of this study was to validate the presence of *A.* *pleuropneumoniae* and *M. hyopneumoniae*, as well as the secondary invaders *P.* *multocida* and *Streptococcus* *suis* in pig herds with a high incidence of pleuritic lesions at slaughter.

## Methods

### Herds and general health status

Four pig herds (A, B, C and D) with continuously high incidences of pleuritis recorded at slaughter (Table 1) were included in the study. All these herds used age segregated rearing with emptying and cleaning of each unit between consecutive batches of growers. The pigs were weaned at a median age of 31 days (range 28–34) and the growers weighted approximately 28 kg when transferred to the fattening unit and around 120 kg at slaughter. Details of herd sizes are included in Table 2.

**Table 1:** Incidence of pleuritis and pneumonia registered at slaughter in four fattening herds with high prevalence of pleuritic lesions recorded at slaughter during 1 year (mean percentage ± standard deviation)

	Pleuritis		Pneumonia			
			Mycoplasma-like		Resembling acute *A.* *pleuropneumoniae*	
	Preceding 4 quarters (%)	Study quarter (%)	Preceding 4 quarters (%)	Study quarter (%)	Preceding 4 quarters (%)	Study quarter (%)
Herd A	32.9 ± 1.0	33.1	0.8 ± 0.8	1.2	0.2 ± 0.1	0.3
Herd B	26.7 ± 5.9	21.5	10.0 ± 2.7	7.5	1.4 ± 1.7	0.8
Herd C	19.3 ± 2.6	20.5	1.1 ± 1.1	0.8	1.5 ± 0.8	0.4
Herd D	26.9 ± 11.5	26.1	3.2 ± 4.4	3.8	0.2 ± 0.2	0.3

The table also shows the prevalence of pleuritis and pneumonia during the quarter of a year when serological profiles were established for individual pigs regarding antibodies to selected bacterial infections. For details, see “Methods” section

**Table 2:** Information about the four herds that participated in the study

	Herd A	Herd B	Herd C	Herd D
Category	Fattening herd	Farrow to finish	Farrow to finish	Farrow to finish
Pigs slaughtered per year	21,000	15,800	6400	22,000
Merchandise of pigs from	1 herd	None	None	None
Vaccination of growers	None	None	None	None
Season studied	Winter	Winter	Winter	Winter
Pigs in unit studied	400	400	400	400

Pigs in Sweden are certified free from African swine fever, Aujeszky’s disease, hog cholera, porcine epidemic diarrhoea, porcine reproductive and respiratory syndrome, transmissible gastroenteritis, and salmonellosis [24].

Endemic viral diseases associated to the respiratory tract include swine influenza that was introduced in 1982. At that time, it caused severe disease outbreaks but today influenza is rarely associated with severe respiratory disease [24]. Porcine respiratory coronavirus (PRCV) entered Sweden in 1987, but has never been associated with severe respiratory disease [24], nor has porcine circovirus type 2 (PCV2). PCV2 was diagnosed for the first time in 1993 in a specific pathogen free (SPF) herd when exudative epidermitis was diagnosed in one batch of piglets [25], which indicated that PCV2 probably had existed earlier in the country.

### Animals and collection of blood samplings

The study was carried out during the winter season in four pig herds with fattening units sized for 400 pigs. All herds applied the “all in–all out” system, and clinical signs of respiratory disease were monitored. On arrival to an empty fattening unit, 10 pens in herd B and 12 pens in herd A, C and D were selected. The pens were evenly distributed within the unit. One pig in each pen was randomly selected and tagged. Blood samples were collected, into tubes without additive, from the tagged pig by jugular venipuncture within the first week after arrival and thereafter every 4th week (week 0, 4 and 8 in all herds, and also week 12 in herd A, C and D). The serum was removed and stored at −20 °C until analysis.

### Registration of pathological lesions in the respiratory tract at slaughter

At slaughter, every pig was inspected by the Swedish Food Administration, a governmental veterinary authority. Lesions in the respiratory tract were registered according to rules set by The Swedish Food Administration (SLVFS 1996:32 and SLVFS 2002:27). Adhesions between lungs and *pleura intercostalis* larger than 10 cm^2^ (a diameter of 3.5 cm) were recorded as pleuritis. Ongoing pneumonic lesions in the cranio-ventral parts of the lungs were recorded as *Mycoplasma*-like pneumonia. Acute pneumonic lesions in other parts of the lung were registered as *A. pleuropneumoniae*-like pneumonia.

### Detection of antibodies to *Actinobacillus pleuropneumoniae*

Antibodies to *A.* *pleuropneumoniae* serotypes 2 and 3 (cross reacting with serotypes 6 and 8) in serum diluted 1/1000 were detected by previously described indirect ELISA systems based on phenol water extracts of the antigens [26]. The absorbance value in serum diluted 1/1000 (A_450_ = 0.5) was used as the limit for defining a positive reaction in both tests.

### Detection of antibodies to *M. hyopneumoniae*

Antibodies to *M.* *hyopneumoniae* in serum diluted 1/40 were detected by a commercial ELISA kit (IDEXX *M. hyo.* Ab test, IDEXX, Westbrook, USA) according the instructions of the manufacturer. The absorbance value in serum diluted 1/40 (A_450_ = 0.4) was used as the limit for defining a positive reaction.

### Detection of antibodies to *P. multocida*

Antibodies to *P.* *multocida* in serum diluted 1/1000 was detected by a previously described indirect ELISA system based on a sonicated whole cell antigen [27]. The absorbance value in serum diluted 1/1000 (A_450_ = 0.5) was used as the limit for defining a positive reaction.

### Detection of antibodies to *S. suis*

Detection of antibodies to *S.* *suis* was made by an indirect ELISA designed for that purpose. The antigen was produced by cultivating *S.* *suis* (strain CCUG 4255) for 18 h at 37 °C on horse blood agar plates. From each plate, the whole growth was harvested in 2 ml PBS without Ca and Mg (pH 7.4; SVA art no 302800) and ultrasonicated (MSE, 60 W ultrasonic disintegrator, Measuring Scientific Equipment Ltd, London, UK) for 5 min per 8 ml solution at 1.3 Ampere with an amplitude of 10 µm. The ultrasonicated solution was centrifuged at 12,000*g* for 20 min at 4 °C (RC2B, Sorvall, Newton, USA). Thereafter, the liquid phase was collected and stored at −20 °C.

Each well in a microtiter plate (Greiner Bio-one, Sigma-Aldrich) was coated with 100 µL of the sonicated antigen diluted 1/10,000 in PBS-T in room temperature for 18 h. Thereafter the microtiter plate was washed three times with PBS-T, and 100 µL serum diluted 1/100 in PBS was added to duplicate wells and the plates were incubated at 37 °C for 1 h. The plates were again washed three times with PBS-T and 100 µL of the conjugate (Protein A-horseradish peroxidase conjugate, Bio-Rad, Richmond, USA) diluted 1/5000 with PBS-T was added to each well and the microtiter plates were stored for 1 h in 37 °C. Then the plates were again washed three times with PBS-T and 100 µL of the substrate with tetra methylbenzidine (TMB, SVANOVA Biotech, Uppsala, Sweden) was added to each well. The reaction was stopped with 100 µL H_2_SO_4_ after 10 min and the absorbance was read at 450 nm by a spectrophotometer (Multiscan MCC/340® MK type II, Labsystem OY, Helsinki, Finland). The results obtained were adjusted to A_450_ = 1.0 for a positive standard serum and absorbance values exceeding 0.5 were regarded as positive reactions, based on the mean absorbance value +2 standard deviations of samples from 72 pigs without clinical signs of *S.* *suis* infection (A_450_ = 0.26 ± 0.12).

### Presentation of serum antibody levels and statistical calculations

**Fig. 2:**
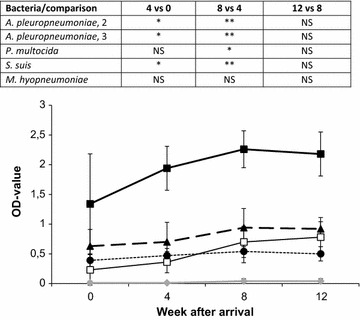
Herd A. Serological profile (mean absorbance ± standard deviation) of 12 pigs repeatedly analyzed during the fattening period at a time when pleuritis was registered in 33.1 % of the pigs at slaughter. The *figure* shows serum levels of antibodies to *A.* *pleuropneumoniae* serotype 2 (*filled square*), serotype 3 (*square*), *P.* *multocida* (*filled triangle*), *S.* *suis* (*filled circle*) and *M.* *hyopneumoniae* (*filled diamond*). Statistical differences to the previous sampling occasion are visualized at the *top* of the figure (NS, P > 0.05; *P < 0.05; **P < 0.01; ***P < 0.001)

**Fig. 3:**
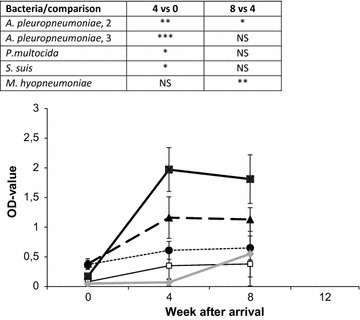
Herd B. Serological profile (mean absorbance ± standard deviation) of 10 pigs repeatedly analyzed during the fattening period at a time when pleuritis was registered in 21.5 % of the pigs at slaughter. The *figure* shows serum levels of antibodies to *A.* *pleuropneumoniae* serotype 2 (*filled square*), serotype 3 (*square*), *P.* *multocida* (*filled triangle*), *S.* *suis* (*filled circle*) and *M.* *hyopneumoniae* (*filled diamond*). Statistical differences to the previous sampling occasion are visualized at the *top* of the figure (NS, P > 0.05; *P < 0.05; **P < 0.01; ***P < 0.001)

**Fig. 4:**
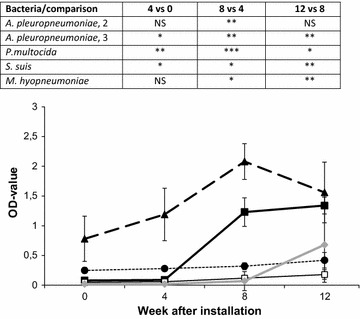
Herd C. Serological profile (mean absorbance ± standard deviation) of 12 pigs repeatedly analyzed during the fattening period at a time when pleuritis was registered in 20.5 % of the pigs at slaughter. The* figure* shows serum levels of antibodies to *A.* *pleuropneumoniae* serotype 2 (*filled square*), serotype 3 (*square*), *P.* *multocida* (*filled triangle*), *S.* *suis* (*filled circle*) and *M.* *hyopneumoniae* (*filled diamond*). Statistical differences to the previous sampling occasion are visualized at the *top* of the figure (NS, P > 0.05; *P < 0.05; **P < 0.01; ***P < 0.001)

**Fig. 5:**
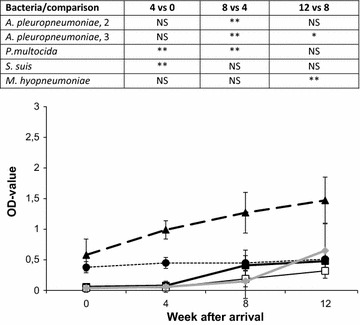
Herd D. Serological profile (mean absorbance ± standard deviation) of 12 pigs repeatedly analyzed during the fattening period at a time when pleuritis was registered in 26.1 % of the pigs at slaughter. The* figure* shows serum levels of antibodies to *A.* *pleuropneumoniae* serotype 2 (*filled square*), serotype 3 (*square*), *P.* *multocida* (*filled triangle*), *S.* *suis* (*filled circle*) and *M.* *hyopneumoniae* (*filled diamond*). Statistical differences to the previous sampling occasion are visualized at the *top* of the figure (NS, P > 0.05; *P < 0.05; **P < 0.01; ***P < 0.001)

The levels of serum antibodies are shown as mean absorbance values with standard deviations in Figs. 2, 3, 4, 5. These figures also show statistical differences in antibody levels between consecutive sampling occasions within herds calculated with the Wilcoxon signed-rank test for matched data. Tables 3, 4, 5, 6 show the number of seropositive and the number of pigs tested at each occasion. These tables include no statistical calculations since the number of pigs tested were too few to allow Chi square analysis, and the variance was too low to allow Fisher’s exact test.

**Table 3:** Herd A

	Week after arrival			
	0	4	8	12
*A. pleuropneumoniae*, type 2	8/12	12/12	12/12	11/11
*A. pleuropneumoniae*, type 3	0/12	2/12	8/12	8/11
*M. hyopneumoniae*	0/12	0/12	0/12	0/11
*P. multocida*	7/12	10/12	12/12	11/11
*S. suis*	1/12	4/12	5/12	4/11

Number of seropositive pigs in 12 pigs repeatedly analysed during the fattening period at a time when pleuritis was registered in 33.1 % of the pigs at slaughter. At week 12, one of the pigs was slaughtered. For details about serum antibody levels, see the corresponding Fig. 2

**Table 4:** Herd B

	Week after arrival			
	0	4	8	12
*A. pleuropneumoniae,* type 2	0/10	10/10	10/10	–
*A. pleuropneumoniae*, type 3	0/10	3/10	3/10	–
*M. hyopneumoniae*	0/10	0/10	4/10	–
*P. multocida*	1/10	10/10	10/10	–
*S. suis*	0/10	8/10	9/10	–

Number of seropositive pigs in 10 pigs repeatedly analysed during the fattening period at a time when pleuritis was registered in 21.5.1 % of the pigs at slaughter. At week 12, all of the pigs were slaughtered. For details about serum antibody levels, see the corresponding Fig. 3

**Table 5:** Herd C

	Week after arrival			
	0	4	8	12
*A. pleuropneumoniae*, type 2	0/12	0/12	12/12	12/12
*A. pleuropneumoniae*, type 3	0/10	0/12	0/12	0/12
*M. hyopneumoniae*	0/12	0/12	0/12	5/12
*P. multocida*	8/12	12/12	12/12	12/12
*S. suis*	0/12	0/12	0/12	2/12

Number of seropositive pigs in 12 pigs repeatedly analysed during the fattening period at a time when pleuritis was registered in 20.5 % of the pigs at slaughter. For details about serum antibody levels, see the corresponding Fig. 4

**Table 6:** Herd D

	Week after arrival			
	0	4	8	12
*A. pleuropneumoniae*, type 2	0/12	0/12	0/12	0/9
*A. pleuropneumoniae*, type 3	0/12	0/12	0/12	0/9
*M. hyopneumoniae*	0/12	0/12	1/12	5/9
*P. multocida*	7/12	12/12	12/12	9/9
*S. suis*	1/12	4/12	3/12	5/9

Number of seropositive pigs in 12 pigs repeatedly analysed during the fattening period at a time when pleuritis was registered in 26.1 % of the pigs at slaughter. At week 12, three of the pigs were slaughtered. For details about serum antibody levels, see the corresponding Fig. 5

## Results

There were no clinical signs of severe respiratory disease during the rearing of the pigs, but the herd prevalence of pleuritic lesions registered at slaughter at that time ranged from 20.5 to 33.1 % (Table 1).

In herd A, the pigs had seroconverted to *A.* *pleuropneumoniae* serotype 2 already on arrival to the fattening units (Fig. 2; Table 3), and the levels of antibodies increased (P < 0.05) during the rearing period. There were also seroreactors to *A.* *pleuropneumoniae* serotype 3, *P.* *multocida* and *S.* *suis* in the herd, but the serum concentrations of antibodies to these microbes remained below A_450_ = 1.0. The herd remained seronegative to *M.* *hyopneumoniae* throughout the rearing period.

In herd B, pigs were seronegative to all microbes tested for on arrival to the fattening unit. After 4 weeks there was a clear seroconversion (P < 0.001) to *A.* *pleuropneumoniae* serotype 2, and also to *P.* *multocida* (P < 0.001) but with a lower concentration of antibodies (Fig. 3; Table 4). There were seroreactors to *S.* *suis*, *A.* *pleuropneumoniae* serotype 3 and *M.* *hyopneumoniae* in the herd, but the serum concentrations of antibodies to these microbes remained below A_450_ = 1.0.

In herd C, pigs were seronegative to *A.* *pleuropneumoniae* serotype 2 and 3, *S* *suis* and *M.* *hyopneumoniae* on arrival. At that time they were seropositive to *P.* *multocida*, and the concentration of antibodies to *P.* *multocida* increased (P < 0.05–0.001) during the two subsequent sampling occasions (Fig. 4; Table 5). Eight weeks after arrival, a clear seroconversion (P < 0.001) to *A.* *pleuropneumoniae* serotype 2 was recorded, whereas antibodies to *M.* *hyopneumoniae*, *A.* *pleuropneumoniae* serotype 3 and *S.* *suis* remained below A_450_ = 1.0.

Also in herd D, pigs were seronegative to *A.* *pleuropneumoniae* serotype 2 and 3, *M.* *hyopneumoniae* and *S.* *suis* on arrival. Regarding *P.* *multocida*, seven out of twelve pigs (58 %) were seropositive on arrival and the antibody concentrations to *P.* *multocida* increased (P < 0.05–0.001) during the two subsequent sampling occasion (Fig. 5; Table 6). In contrast, the antibody concentrations to the other agents remained below A_450_ = 1.0 throughout the rearing period.

## Discussion

The results obtained confirmed a low pathogen load of *M.* *hyopneumoniae*, which concurred well with the decreased incidence of pneumonic lesions recorded at slaughter following the implementation of strict age segregated rearing systems (all in–all out) in Sweden during the 1990s [21, 22] as shown in Fig. 1. It could of course, be argued that pulmonary lesions due to *M.* *hyopneumoniae* heal with time [28, 29], and therefore, infections gained during the early rearing period could escape detection at slaughter. However, the low levels (A_450_ < 1.0 in all herds) of antibodies to *M.* *hyopneumoniae* recorded show that the registrations of pneumonia were correct. Still, the slight increase of serum antibodies to *M. hyopneumoniae* at the end of the rearing period in herds B, C and D indicate the presence of *M.* *hyopneumoniae* in these herds, and that should not be neglected. The global market weight of pigs varies from around 80–180 kg, and is at present around 120 kg in Sweden, which is reached at an age of 6–7 months. If the market weight increase also the rearing period will be prolonged with more days at risk for each pig, which may pave the way for clinical signs of *M.* *hyopneumoniae*. Although the pathogen load differs between Sweden and Italy, it is notable that *M.* *hyopneumoniae*-like lesions were recorded in 2268 out of 4889 pigs (45.4 %) slaughtered at an age of 9–10 months in Italy [30].

Traditionally *A.* *pleuropneumoniae* has been strongly associated with pleuritis [6], and the capability of *A.* *pleuropneumoniae* to induce pleuritis was visualized by herd A in this study where the serological profile suggested *A.* *pleuropneumoniae* serotype 2 to be the sole bacterial cause of the high incidence of pleuritic lesions recorded at slaughter, although a possible influence from viral infections [15, 16] not can be excluded. However, the high levels of serum antibodies to *A.* *pleuropneumoniae* (A_450_ > 1.5) and low levels of antibodies to other bacteria (A_450_ < 1) was concluded to illustrate a classic serological pattern (Fig. 2).

Still, the results obtained in herds B, C and D in this study suggest that pleuritis in pigs could be a multifactorial syndrome rather than being linked to a single specific infection as also has been described by others [11–14, 18].

The synergistic influence of a secondary invader was clear in herd B, where high levels (A_450_ > 1.5) of antibodies to *A.* *pleuropneumoniae* serotype 2 were followed by significant levels (A_450_ > 1.0) of antibodies to *P.* *multocida*. This suggested a strong influence from *P.* *multocida* as also has been suggested earlier [17, 18, 31]. Also the levels of antibodies to *M.* *hyopneumoniae* and *S.* *suis* increased to some extent during the end of the rearing period However, as the antibody levels to these microbes remained at low levels (A_450_ < 1.0) their influence on the lung score were considered to be less significant. Thus, the serological pattern in herd B suggested *A.* *pleuropneumoniae* serotype 2 to be the main cause of the pleuritic lesions, but these lesions may have been amplified by subsequent secondary infections—especially with *P.* *multocida*.

In herd C the serological response to *P.* *multocida* was strong (A_450_ > 1.5) and preceded that to *A.* *pleuropneumoniae* serotype 2, and the influence of *P.* *multocida* therefore should be regarded as even more significant in this herd. Still, the increasing levels of antibodies to *A.* *pleuropneumoniae* serotype 2 at the end of the rearing period suggested an influence of *A.* *pleuropneumoniae* also in this herd, and it is notable that the levels of antibodies to *M.* *hyopneumoniae* increased slightly during the end of the rearing period. As *P.* *multocida* is regarded to be a secondary invader, something else than *A.* *pleuropneumoniae* or *M.* *hyopneumoniae* ought to have paved the way for that microbe. Although this remain undiagnosed in the present study, the frequent demonstrations of interferon-α in serum of fattening pigs during the first week after allocation [15, 16] indicate that viral infections may be precursors to *P.* *multocida* and the frequent findings of different virus in pigs using novel techniques [32] support that hypothesis. The early infections with *P.* *multocida* may by themselves not necessarily have induced pleuritic lesions, but obviously the infection with *P.* *multocida* already was established as the pigs became infected with *A.* *pleuropneumoniae* and colonies of *P.* *multocida* already on site may have amplified the effect of the subsequent *A.* *pleuropneumoniae* infection.

The serological pattern in herd D suggested a minor impact of *A. pleuropneumoniae* despite the high frequencies of pleuritic lesions recorded at slaughter. The mean concentration of antibodies to *A.* *pleuropneumoniae* serotype 2 and 3 remained below the cut off-value during the entire rearing period. Instead, pigs were seropositive to *P.* *multocida* already on arrival to the fattening unit and the level of serum antibodies to *P.* *multocida* increased throughout the rearing period in absence of antibodies to the other microbes. This clearly indicated that pleuritic lesions may evolve at high frequencies also in absence of *A.* *pleuropneumoniae*, as also has been suggested by others [33]. Likewise, no correlation between *A.* *pleuropneumoniae* and pleuritis at individual level was seen in herds with low incidences of pleuritic lesions recorded at slaughter [34]. Instead seroconversion to *M.* *hyopneumoniae* during the early fattening period was related to pleuritis at an individual level in such herds, which indicated an influence of secondary infections [34]. Therefore, the common presence of serum antibodies to *P.* *multocida* is of interest. However, in the present study, *P.* *multocida* was associated with a high prevalence of pleuritic lesions recorded at slaughter in absence of *M.* *hyopneumoniae*. Thereby, the true initial cause for these lesions still remains unknown and warrants further investigations. Since viral infections repeatedly has been demonstrated during the early fattening period [15, 16] viral infections may well have preceded the serological response to *P.* *multocida*.

## Conclusion

Pleuritic lesions registered at slaughter ranged from 20.5 to 33.1 % in the four herds. High levels of serum antibodies to *A. pleuropneumoniae* and *P. multocida*, either alone or in combination, were seen. Pigs in this study seroconverted to *M.* *hyopneumoniae* late during the rearing period (herd B–D), or not at all (herd A), confirming a positive effect of age segregated rearing in preventing or delaying infections with *M.* *hyopneumoniae.* The results obtained highlight the necessity of diagnostic investigations to define the true disease pattern in herds with a high incidence of pleuritic lesions.

## References

[1] Maré CJ, Switzer WP (1965). New species: *Mycoplasma* *hyopneumoniae*, the causative agent of virus pig pneumonia. Vet Med.

[2] Goodwin RFW, Pomerov AP, Whittlestone P (1965). Production of enzootic pneumoniae in pigs with a mycoplasma. Vet Rec.

[3] Thacker EL, Minion FC, Zimmermann JJ, Karriker LA, Ramirez A, Schwartz KJ, Stevenson G (2012). Mycoplasmosis. Diseases of swine.

[4] Shope RE, White DC, Leidy G (1964). Porcine contagious pleuropneumoniae II. Studies of the pathogenicity of the etiological agent *Haemophilus* *pleuropneumoniae*. J Exp Med.

[5] Bieberstein EL, Gunnarsson A, Hurvell B (1976). Cultural and biochemical criteria for the identification of *Haemophilus* culture from swine. Am J Vet Med Assoc.

[6] Gottschalk M, Zimmermann JJ, Karriker LA, Ramirez A, Schwartz KJ, Srevenson G (2012). Actinobacillosis. Disieases of swine.

[7] Betts AO (1952). Respiratory disease of pigs. V. Some clinical and epidemiological aspects of virus pneumonia of pigs. Vet Rec.

[8] Brockmeier SL, Palmer MV, Bolin SR (2000). Effects of intranasal inoculation of porcine reproductive and respiratory syndrome virus, *Bordetella* *bronchiseptica*, or a combination of both organisms in pigs. Am J Vet Res.

[9] Brockmeier SL, Loving CL, Nicholson TL, Palmer MV (2008). Coinfection of pigs with porcine respiratory coronavirus and *Bordetella* *bronchiseptica*. Vet Microbiol.

[10] Loving CL, Brockmeier SL, Vincent AL, Palmer MV, Sacco RE, Nicholson TL (2010). Influenza virus coinfection with *Bordetella* *bronchiseptica* enhances bacterial colonization and host response exacerbating pulmonary lesions. Microb Pathog.

[11] Nicholson TL, Brockmeier SL, Loving CL, Register KB, Kehrly EK, Shore SM (2014). The *Bordetella* *bronchiseptica* type III secretion system is required for persistence and diseases severity but not transmission in swine. Infect Immun.

[12] Eze JI, Correia-Gomes C, Borobia-Belsue J, Tucker AW, Sparrow D, Strachan DW, Gunn GJ (2015). Comparison of respiratory disease prevalence among voluntary monitoring systems for pig health and welfare in the UK. PLoS ONE.

[13] Little TWA (1975). Respiratory disease in pigs: a study. Vet Rec.

[14] Hansen MS, Pors SE, Jensen HE, Bille-Hansen V, Bissgaard M, Flachs EM, Nielsen OL (2010). An investigation of the pathology and pathogens associated with porcine respiratory disease complex in Denmark. J Comp Path.

[15] Artursson K, Wallgren P, Alm GV (1989). Appearance of interferon-α in serum and signs of reduced immune functions in pigs after transport and installation in a fattening farm. Vet Immunol Immunopath.

[16] Wallgren P, Artursson K, Fossum C, Alm GV (1993). Incidence of infections in pigs bred for slaughter revealed by elevated serum levels of interferon and development of antibodies to *Mycoplasma* *hyopneumoniae* and *Actinobacillus* *pleuropneumoniae*. J Vet Med B.

[17] Bölske G, Martinsson K, Persson N, Nielsen NC, Høgh P, Bille N (1980). The incidence of mycoplasma and bacteria from lungs of swine with enzootic pneumonia in Sweden. International pig veterinary society congress.

[18] Ciprian A, Pijoan C, Cruz T, Camacho J, Tortora J, Colmenares G, Lopez-Revilla R, De la Garza M (1988). *Mycoplasma hyopneumoniae* increase the susceptibility of pigs to experimental *Pasteurella multocida* pneumonia. Can J Vet Res.

[19] Wallgren P (2009). First out to ban feed additives in 1986. Veterinary challenges in the Swedish pig production. Part I. Use of antimicrobials and respiratory diseases. Pig J.

[20] Wallgren P (2009). First out to ban feed additives in 1986. Veterinary challenges in the Swedish pig production. Part II. Intestinal and miscellaneous diseases. Pig J.

[21] Holmgren N, Lundeheim N (2002). Rearing systems and health of pigs in Sweden. Svensk VetTidn.

[22] Wallgren P, Lindberg M, Sjölund M, Karlsson Frisch K, Ericsson Unnerstad H (2015). Antimicrobial resistance in *Actinobacillus* *pleuropneumoniae* and *Pasteurella* *multocida* isolated from the respiratory tract of pigs in Sweden. Svensk VetTidn.

[23] Wallgren P, Aspán A (2009). *Actinobaccillus* *pleuropneumoniae.* A comparison of Swedish isolates of serotype 2 and 5 over time. Pig J.

[24] Anonymous. Surveillance of infectious diseases in animals and humans in Sweden 2014. National Veterinary Institute (SVA), Uppsala. SVA:s rapportserie 31. ISSN 1654-7098. http://www.sva.se.

[25] Wattrang E, McNelly F, Allan GM, Greko C, Fossum C, Wallgren P (2002). Exudative epidermitis and porcine circovirus-2 infection in a Swedish SPF herd. Vet Microbiol.

[26] Wallgren P, Persson M (2000). Relationship between the amounts of antibodies to *Actinobacillus* *pleuropneumoniae* serotype 2, detected in blood serum and in fluids collected from muscles of pigs. J Vet Med B.

[27] Sjölund M, Zoric M, Persson M, Karlsson G, Wallgren P (2011). Disease patterns and immune responses in the offspring to sows with high or low antibody levels to *Actinobaccillus* *pleuropneumoniae* serotype 2. Res Vet Sci.

[28] Noyes EP, Feeny DA, Pijoan C (1990). Comparison of the effect of pneumonia detected during lifetime with pneumonia detected at slaughter on growth in swine. J Am Vet Med Assoc.

[29] Wallgren P, Beskow B, Fellström C, Renström HML (1994). Porcine lung lesions at slaughter and their correlation to the incidence of infections by *Mycoplasma* *hyopneumoniae* and *Actinobacillus* *pleuropneumoniae* during the rearing period. J Vet Med B.

[30] Merialdi G, Dottori M, Bonilauri P, Luppi A, Gozio S, Pozzi P, Spaggiaria B, Martelli P (2012). Survey of pleuritis and pulmonary lesions in pigs at abattoir with focus on the extent of the conditions and herd risk factors. Vet J.

[31] Pijoan C, Fuentes M (1987). Severe pleuritic associated with certain strains of *Pasteurella* *multocida* in swine. J Am Med Assoc.

[32] Blomström AL, Belák S, Fossum C, McKillen J, Allan G, Wallgren P, Berg M (2009). Detection of a novel porcine boca-like virus in the background of porcine circovirus type 2 induced post weaning multisystemic wasting syndrome. Vir Res.

[33] Fablet C, Marois C, Dorenlor V, Eono F, Eveno E, Jolly JP, Le Devendec L, Kobisch M, Madec F, Rose N (2012). Bacterial pathogens associated with lung lesions in slaughter pigs from 125 herds. Res Vet Sci.

[34] Holmgren N, Lundeheim N, Wallgren P (1999). Infections with *Mycoplasma* *hyopneumoniae* and *Actinobacillus* *pleuropneumoniae* in fattening pigs. Influence of piglet production system and influence on production parameters. J Vet Med B.

